# *Cistanche deserticola* Polysaccharide Ameliorates Cyclophosphamide-Induced Splenic Immunosuppression in Mice: Integrated Transcriptomic and Proteomic Analyses of Immune Modulation

**DOI:** 10.3390/antiox15081048

**Published:** 2026-08-21

**Authors:** Baotang Zhao, Faqin Tao, Shengfang Wang, Guofeng Li, Mingze Li, Yulong Huang

**Affiliations:** 1 College of Food Science and Engineering, Gansu Agricultural University, Lanzhou 730070, China; 2 College of Life Science, Northwest Normal University, Lanzhou 730070, China; faqintao@126.com; 3 Institute of Agricultural Products Storage and Processing, Gansu Academy of Agricultural Sciences, Lanzhou 730070, China

**Keywords:** *Cistanche deserticola* polysaccharide, immunity regulation, spleen, transcriptome–proteome integration

## Abstract

*Cistanche deserticola* polysaccharide (CDP) exhibits pleiotropic bioactivities, yet its splenic-protective profile remains incompletely defined. Here, male ICR mice were challenged with cyclophosphamide (CTX) to establish an immunosuppressed model and concurrently treated with CDP. By integrating functional assays, splenic histopathology, and transcriptomic and proteomic analyses, we show that CDP dose-dependently restores splenic mass, rescues white-pulp atrophy, and suppresses megakaryocytic hyperplasia. Functionally, CDP rebalances pro-/anti-inflammatory cytokines, scavenges splenic ROS/MDA, and potentiates GSH-Px/SOD antioxidant capacity versus CTX alone. Multi-omics convergence (3103 DEGs; 1387 DEPs) delineated a CDP-distinctive signature related to innate immune recognition, oxidative stress buffering, and protease/ion-transport modules. Notably, transcriptional enrichment of neutrophil extracellular trap (NET)-associated proxies—synergized with NOD-like receptor/IL-17 signaling—suggests a putative innate-priming mechanism warranting functional validation, rather than confirmed pathway activation. Collectively, CDP mitigates CTX-induced splenic injury primarily through coupled redox restoration and transcriptional-level immune modulation.

## 1. Introduction

*Cistanche deserticola Ma*, a traditional medicinal and food plant, contains polysaccharides [1], polyphenols, volatile compounds, cyclic allyl ether terpenes, and phenylthiols [2] and has attracted much attention mainly because of its rich *Cistanche deserticola* polysaccharide (CDP) with significant immunomodulatory activities [3]. CDP exhibits a wide range of pharmacological activities intertwined with its immunomodulatory action, such as anti-aging, antioxidant, and anti-carcinogenic effects, among other biological functions, and all of these are achieved by enhancing the body’s immune function [4]. Immunomodulatory activity is one of the most important biological functions of CDP, and the regulation of immune function by CDP occurs through multiple pathways, multiple interactions, and multiple targets [5]. Studies have shown that the immunological activation of dendritic cells in mice is associated with the activation of the TLR4/NF-κB signaling pathway, which reflects the imbalance in the gut microbiota caused by cyclophosphamide and has significant potential for modulating immunity [6]. Jiang et al. [7] jointly investigated, in a combination of proteomics and intestinal flora, another relevant polysaccharide, DZMP. By establishing a cyclophosphamide-immunosuppressed mouse model, it was found that DZMP could protect the immune organs of immunosuppressed mice, increase the content of immune factors in mice, enhance the antioxidant capacity, change the content of SCFAs [8], up-regulate the expression of FcγRIII and PKC-α proteins, promote energy metabolism, and maintain intestinal health, thus playing an immunomodulatory role. By strengthening immune organ function, modulating immune cytokines and alleviating oxidative stress damage—combined with the remodeling of the gut microbiota, optimization of the intestinal environment and regulation of the key PI3K-Akt signaling pathway—Liang et al. effectively improved the immunosuppressed state in mice, thereby providing a basis for the development of black cumin seed polysaccharides as functional foods or immunomodulators [9]. Shan et al. found that polysaccharides of yellow seedpods (PCP-80%) triggered the mechanism of immune activation [10], which could effectively regulate the immune responses of macrophages, activating JAK-STAT, MAPKs and the NF-κB signaling pathway [11].

Multi-omics offers a comprehensive understanding of biological functions, molecular mechanisms and interactions underlying disease and has the potential to transform the field of drug discovery and drive the emergence of innovative and effective therapeutic approaches [12]. A transcriptomic–metabolomic study has revealed that corn starch polysaccharides (CSPs) promote colorectal cancer (CRC) by altering the gut microbiota, disrupting metabolic pathways (such as choline and arachidonic acid metabolism) and enhancing inflammatory signaling (such as PI3K-Akt and NF-κB). These findings provide new insights into the pro-inflammatory potential of certain dietary polysaccharides [13]. DOP, a polysaccharide derived from Dendrobium officinale, induces cell-cycle arrest and apoptosis via the PI3K-AKT and NF-κB signaling pathways and shows potential as a targeted therapy for T-cell acute lymphoblastic leukemia [14]. Plant seed polysaccharides exert immunomodulatory effects via the TLR4/NF-κB, PI3K-Akt and MAPK pathways [15]. The polysaccharide extracted from Dongsheng alkaloids, CPAP, promotes the enrichment of Lactobacillus in the gut, regulates purine metabolism to activate T-cell-mediated anti-tumor immunity, and promotes neutrophil degranulation to alleviate tumor inflammation. By increasing oxidative stress to induce tumor cell apoptosis, it provides support for dietary adjuvants targeting immune-deficiency-related diseases [16]. This study aimed to define the splenic-protective phenotype of medium-dose CDP in a CTX model via integrated histopathology, redox profiling, and transcriptomic–proteomic convergence.

## 2. Materials and Methods

### 2.1. Materials and Reagents

A total of 70 KM mice (Lanzhou Veterinary Research Institute) were selected. The following materials were used: prepared Cistanche polysaccharide (CDP-D2-N1); cyclophosphamide (Shanghai Yuanye Biotechnology Co., Ltd., Shanghai, China); IL-1, IL-2, IL-6, TNF-α, and IFN-γ (Beijing Solarbio Science & Technology Co., Ltd., Beijing, China); CAT, SOD, MDA, GSH, GSH-PX, and T-AOC (Nanjing Jiancheng Bioengineering Institute, Nanjing, China); IgA ELISA, IgM ELISA and IgG ELISA Kits (Shanghai Yilai Biotechnology Co., Ltd., Shanghai, China).

### 2.2. Extraction of CDP-D2-N1

Cistanche polysaccharides were extracted using ultrasound-assisted hot water extraction; following separation and purification, the most potent fraction was selected and named CDP-D2-N1. This is an amorphous structure with a molecular weight of 127.784 kDa, composed primarily of galacturonic acid (ManA), galactose (Gal) and arabinose (Ara). In subsequent animal experiments, CDP-D2-N1 will be referred to simply as CDP.

### 2.3. Animals and Experimental Design

Following a 3-day acclimatization period and feeding, the mice were divided into 7 groups of 10 mice each based on body weight [17]. One group was designated as the normal control group (NC), with each mouse receiving daily injections of saline (0.85%, 20 mL/kg) for 9 consecutive days. The remaining six groups of mice were each injected with CTX (100 mg/kg) on days 1–2; the second group was designated as the cyclophosphamide group (CTX), and these mice were gavaged with an equal volume of saline on days 3–9. The levamisole hydrochloride group (MC group, 100 mg/kg/day), the astragalus polysaccharide group (HQP group, 200 mg/kg/day), and the low-, medium- and high-dose-CDP treatment groups (CDP-L, CDP-M, and CDP-H) were administered 100 mg/kg/day of MC and HQP via oral gavage on days 3–9. The CDP-L, CDP-M and CDP-H groups were administered 100 mg/kg/day, 200 mg/kg/day and 400 mg/kg/day, respectively. On day 10, the mice were fasted but allowed free access to water; blood was collected from the orbital vein; the mice were decapitated and weighed, and their spleens were removed and weighed to calculate the spleen index. Following the method described by Li et al. [18], the supernatant of the resulting tissue homogenate was used to determine enzyme activity and cytokine levels. Spleens were harvested as described. For bulk transcriptomic and proteomic profiling, we purposely selected CDP-M as the representative treatment arm, because pilot pharmacodynamics (spleen index, IL-2/IL-6 ratio, and antioxidant restoration) identified CDP-M as the optimal immunomodulatory dose without overt toxicity. Omics analyses therefore compared three biologically independent sub-groups (*n* = 3 randomly selected spleens per arm from the original 10-animal cohorts), normal control (NCP), cyclophosphamide-only model (CTXP), and CDP-M intervention (CDPP), minimizing batch effects while capturing the core mechanistic axis. All animal experiments were conducted in rigorous compliance with locally approved experimental regulations (Permit No.: SYXK (Gan) 2025-0011) and the “Law of the People’s Republic of China on the Management of Laboratory Animals”.

### 2.4. Spleen Functional Assays (Index, Cytokines, and Oxidative Stress)

The spleen index was calculated as (spleen wet weight, mg/body weight, g) × 100. The following procedure was performed: Pre-treat the samples according to the instructions for each respective kit. Remove the spleen tissue from the −80 °C freezer, and place it in an ice-water bath to thaw. Rinse away residual blood with pre-chilled PBS (pH 7.4), weigh the tissue, and then mince it. Homogenize the tissue with an appropriate volume of PBS on ice. Centrifuge the homogenate at 5000× *g* for 10 min, and collect the supernatant for analysis. Following this procedure, quantitative assays for TNF-α, IFN-γ, IL-1, IL-6 and IL-2 were performed in sequence according to the respective protocol instructions. Levels of TNF-α, IFN-γ, IL-1β, IL-6, and IL-2 in splenic homogenates were quantified by commercial ELISA kits (protocols per kit inserts). Oxidative stress markers (MDA by the TBA method; ROS by DCFH-DA probe) and antioxidant enzymes (GSH-Px, GR, CAT, SOD, GSH/GSSG, and T-AOC) were assayed using a Beckman DU730 spectrophotometer (Beckman Coulter, Brea, CA, USA). These standardized workflows were adapted from our previously published, methodologically identical protocols [19]; core procedural steps are recapitulated here to ensure standalone reproducibility, with reagent specifics and validation curves fully documented in Ref. [19].

### 2.5. Histopathological Section Observation

Spleen tissue samples of about 1 cm^3^ were taken and quickly fixed in 4% paraformaldehyde (PFA; pH 7.4) solution for 24 h. Subsequently, tissue trimming, gradient ethanol dehydration, xylene transparency, paraffin embedding and 4 μm thickness sectioning were performed sequentially. After the sections were stained with hematoxylin–eosin (H&E), they were observed using a Nikon Eclipse Ci-L orthostatic light microscope, and a 20× objective lens (total magnification ×200) was selected for image acquisition [19]. A transmitted light source was uniformly used during imaging, Koehler illumination was adjusted to ensure uniform brightness in the field of view, and white balance was calibrated to avoid chromatic aberration. Three representative fields of view (cortical, limbic and medullary regions) were selected for each section to ensure complete coverage of the imaging area.

### 2.6. Transcriptomic Analysis

Spleen tissues from the CDP-M (200 mg/kg) group, CTX model, and NC group were snap-frozen at −80 °C after liquid nitrogen snap-freezing [19]. Total RNA was extracted using TRIzol^®^ reagent (Invitrogen, Carlsbad, CA, USA) and tested for purity by a Nanodrop ND-2000 (Thermo Fisher Scientific, Waltham, MA, USA), and RNA integrity was assessed by an Agilent Bioanalyzer 4150 (Agilent Technologies, Santa Clara, CA, USA). QC-qualified samples were used to construct double-ended sequencing libraries using the AB clonal mRNA-seq Lib Prep Kit (ABclonal Biotechnology Co., Ltd., Wuhan, Hubei, China): briefly, 1 μg of total RNA was fragmented by oligo(dT)-enriched magnetic beads; double-stranded cDNA was synthesized with random primers, ligated to Illumina junctions and amplified by PCR; and the libraries were QC-qualified and amplified on an Illumina Nova Seq. After library quality control, the libraries were sequenced on an Illumina Nova Seq 6000/MGISEQ-T7 platform (Illumina Inc., San Diego, CA, USA/MGI Tech Co., Ltd., Shenzhen, Guangdong, China) at 150 bp (≥6 Gb of clean data per sample). After the raw data were quality-controlled by Fast QC, low-quality reads (>60% of bases with Phred score ≤ 25 or >5% of N ratio) were removed by Trimoraic and compared with the mouse reference genome GRCm39 by HISAT2, and gene expression (FPKM value) was calculated by using Feature Counts. Differentially expressed genes (DEGs) were screened by DESeq2 (|log2FC| > 1 and Padj < 0.05), and GO functional enrichment and KEGG pathway analysis were performed by cluster Profiler (*p* < 0.05), with a focus on annotation of Toll-like receptor, NF-κB, and cytokine signaling pathways. To mine potential immune regulatory networks, protein–protein interaction (PPI) networks were constructed based on the STRING database (v12.0) (confidence level > 0.7), and the core module was visualized by Cystoscope; the global enrichment trend of the immune function-related gene set was assessed using GSEA analysis (FDR < 0.25) in parallel. In addition, transcription factors of DEGs were annotated using Animal TFDB 4.0, and variable shear events were analyzed by rMATS.

### 2.7. Proteomic Analysis

#### 2.7.1. Protein Extraction and Peptide Digestion

An appropriate volume of SDT lysis buffer was added to each sample to extract proteins, which were then quantified using the BCA method. From each sample, 15 µg of protein was taken and added to an appropriate volume of 5× loading buffer, followed by heating in a boiling water bath for 5 min. SDS-PAGE electrophoresis was performed, and the gels were stained with Coomassie Blue R-250. An appropriate amount of protein was taken from all samples, pooled to form a pool sample, and used as a QC sample. All samples were digested with trypsin using the Filter-Aided Proteome Preparation (FASP) method. The digested peptides were desalted and freeze-dried and then resuspended in 40 µL of 0.1% formic acid solution, and the peptide concentration was determined by measuring the OD280. An appropriate amount of iRT standard peptides was added to the digested peptides of each sample, and DIA mass spectrometry analysis was performed using an Astral high-resolution mass spectrometer (Thermo Fisher Scienific, Waltham, MA, USA).

#### 2.7.2. Bioinformatics Analysis

The quantitative data for the target protein set were normalized. The Complex heatmap R package (Bioconductor, version 2.18.0) was used to perform clustering across both the sample and protein expression dimensions. Mfuzz software (Bioconductor, version 2.60.0) was employed to perform fuzzy c-means clustering analysis, dividing the data into distinct expression modules based on expression trends to generate different clusters. Subcellular localization was predicted using the CELLO method (National Chiao Tung University, Hsinchu, Taiwan, China; http://cello.life.nctu.edu.tw/) (accessed 19 August 2025), and protein domain (https://pfam.xfam.org/, accessed on 19 August 2025) analysis was conducted using the Pfams database. Blast2GO was utilized to perform GO annotation on the target protein set, whilst KAAS software (Ver. 2.1, KEGG Automatic Annotation Server, Kyoto University, Kyoto, Japan; https://www.genome.jp/, accessed on 19 August 2025) was employed to perform KEGG pathway annotation on the same set, resembling the detection method described by Xin et al. [20].

### 2.8. Statistical Analysis

Data were analyzed statistically using origin 9.0 and SAS 8.0 software to analyze the experimental results. Data are expressed as means ± SD. Prior to inference, normality was assessed by the Shapiro–Wilk test and homogeneity of variances by Levene’s test. For multi-group comparisons across all seven experimental arms, parametric data were analyzed by one-way ANOVA with Tukey’s honest significant difference post hoc test; non-normally distributed variables used Kruskal–Wallis test with Dunn’s correction for multiple comparisons. Pearson correlation supported mRNA–protein concordance.

## 3. Results

### 3.1. Effect of CDP on Splenic Index in Immunocompromised Mice

**Figure 1:**
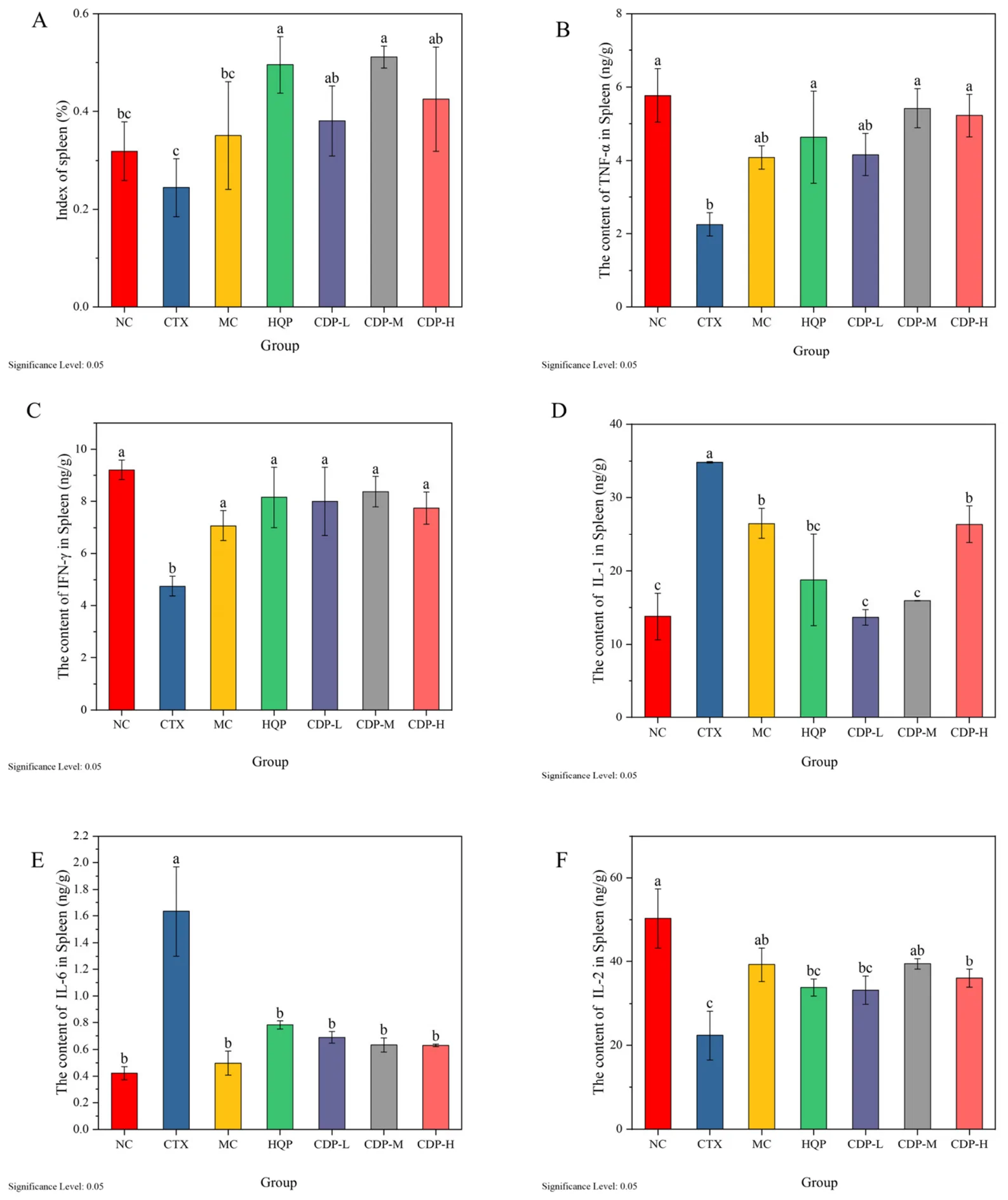
Effects of CDP on changes in splenic index (**A**) and TNF-α (**B**), IFN-γ (**C**), IL-1 (**D**), IL-6 (**E**), and IL-2 (**F**) levels in immunosuppressed mice. Different lowercase letters above bars indicate statistically significant differences between groups. Groups sharing the same letter(s) show no significant difference, while groups marked with different letters indicate a statistically significant difference (*p* < 0.05).

As shown in Figure 1A, the spleen index of mice in the CTX group was significantly lower (*p* < 0.05) than that in the blank group, and this result proves that the immune organs of the mice in the model group were severely damaged. Compared with the CTX group, the spleen index of mice after CDP gavage was significantly higher (*p* < 0.05), close to that of the NC group, indicating that CDP could increase the immunomodulatory ability by stimulating the spleen of mice. In addition, in mice treated with different doses of CDP, we found that treatment stimulated the growth and development of the mouse thymus and spleen in a dose-dependent manner. As can be seen from the figure, the spleen indices of mice in the HQP and CDP groups were significantly higher than those in the NC group (*p* < 0.01), whereas the spleen indices of mice in the group administered CTX via injection were significantly higher (*p* < 0.05) compared with the other groups. The results indicate that CDP could significantly and quickly increase the low splenic index caused by immune injury, thus counteracting the damage to the hematopoietic system caused by CTX in mice.

### 3.2. Effects of CDP on Cytokines in Spleen of Immunocompromised Mice

#### 3.2.1. Effects on TNF-α and IFN-γ Contents in Spleen of Immunocompromised Mice

As can be seen in Figure 1B,C, the level of tumor necrosis factor (TNF)-α in the spleen of the model group was higher compared with the blank control group, and the difference was significant (*p* < 0.05), while the TNF-α levels in both control groups with CTX were not significantly different compared with the NC group. This indicates that CDP does not cause the spleen to produce excessive TNF-α or cause the organism to produce malignant fluid. However, in mice with immune damage caused by CTX, TNF-α promotes the differentiation of myeloid cells into macrophages and improves the immune function of mice. TNF-α is a mononuclear factor produced by monocytes and macrophages. CDP can reverse the inhibitory effects of CAT on immune cells, such as T cells, NK cells, macrophages, and LAK cells [21]. Mice in the model group had outstandingly lower IFN-γ levels due to CTX. After CDP treatment was given to CTX-inhibited immunocompromised mice, IFN-γ levels gradually returned to near-normal levels.

#### 3.2.2. Effects on IL-1, IL-2 and IL-6 Contents in Spleen of Immunocompromised Mice

As can be seen in Figure 1D–F, the levels of IL-1 and IL-6 in the spleen of mice in the model group were significantly higher (*p* < 0.05), and the levels of IL-2 were significantly lower (*p* < 0.05) as a result of the action of CTX, compared with mice in the control group. The levels of IL-1 and IL-6 in the spleen tissues of mice in the CDP group were reduced relative to the model group, and the difference was significant (*p* < 0.05). Compared with the CTX group, on the other hand, IL-2 content was elevated, and the increase in IL-2 content in the spleen of mice in the middle-dose and high-dose groups reached a significant level (*p* < 0.05). IL levels in the spleen of mice in the CDP gavage group did not differ significantly from those in the three dose groups (*p* > 0.05). CDP can restore levels of IL-1, IL-6, and IL-2 in the thymus of immunocompromised mice, indicating that CDP promotes T-cell activation and augments the immune response.

### 3.3. Effect of CDP on the Oxidative Stress State in Spleen of Immunocompromised Mice

**Figure 2:**
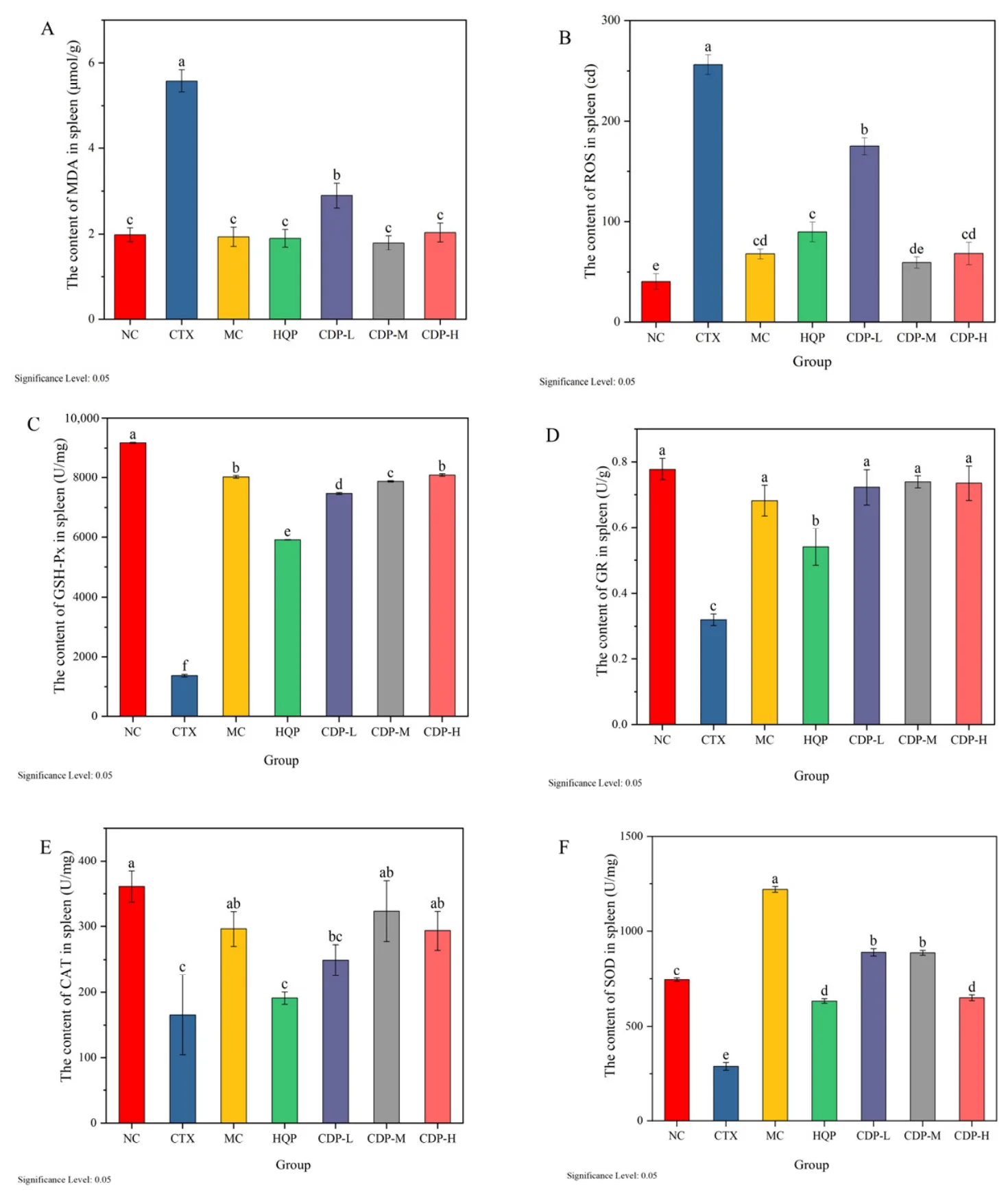
Effects of CDP on MDA (**A**), ROS (**B**), GSH-Px (**C**), GR (**D**), CAT (**E**) and SOD (**F**) contents in spleen of immunosuppressed mice. Groups sharing the same letter(s) show no significant difference, while groups marked with different letters indicate a statistically significant difference (*p* < 0.05).

As can be seen from Figure 2A,B, the MDA content and ROS levels in the spleen of CTX mice were higher than those in the other groups, with a significant difference (*p* < 0.05), whereas the differences between the rest of the groups and NC mice were not significant (*p* > 0.5). The polysaccharide was able to resist lipid oxidation after CDP gavage in mice immunocompromised by CTX, while MDA content and ROS level decreased more significantly due to the faster renewal of splenocytes.

### 3.4. Effects of CDP on Oxidoreductase in Spleen of Immunocompromised Mice

#### 3.4.1. Effects on Levels of GSH-Px and GR in Spleen of Mice

As can be seen in Figure 2C,D, GSH-Px and GR activities in the spleen of mice in the model group were decreased compared with those in the NC group, and the differences were significant (*p* < 0.05). GSH-Px and GR activities in the spleen of mice in all groups were significantly enhanced after CDP gavage (*p* < 0.05). There were no significant differences between the GR activities in the spleen of mice in the NC group and mice in groups with different dosages of CDP (*p* > 0.05), and there were no significant differences between the mice in the CDP gavage groups, whereas there was a significant difference between splenic GSH-Px activity in mice in the gavage group and that in the NC group (*p* < 0.05) and between the mice in the CDP groups (*p* < 0.05). The results indicate that CDP up-regulated splenic GR activity and promoted GSH regeneration and that GSH-Px activity was synchronously elevated, thus improving CTX-induced immune injury and restoring neutrophil phagocytosis rate and T-cell ratio.

#### 3.4.2. Effects on Contents of CAT and SOD in Spleen of Mice

Splenic SOD and CAT responded divergently to CDP intervention (Figure 2E,F). Both enzymes were suppressed in CTXP mice versus normal controls (NCs), confirming CTX-induced redox collapse. CDP-M/CDP-H restored CAT activity to levels significantly above CTXP (*p* < 0.05) yet remained below the NC baseline (non-significant vs. NCs for CDP-M, *p* > 0.05), whereas SOD activity exceeded even NC values (∼1.3-fold, *p* < 0.05). This enzyme-specific redox profile indicates that CDP does not uniformly normalize all antioxidant arms; rather, SOD is preferentially up-regulated—or CAT demand is attenuated—by polysaccharide-direct free-radical quenching that lessens steady-state H_2_O_2_ burden.

#### 3.4.3. Effects on Contents of GSH and GSSG in Spleen of Mice

**Figure 3:**
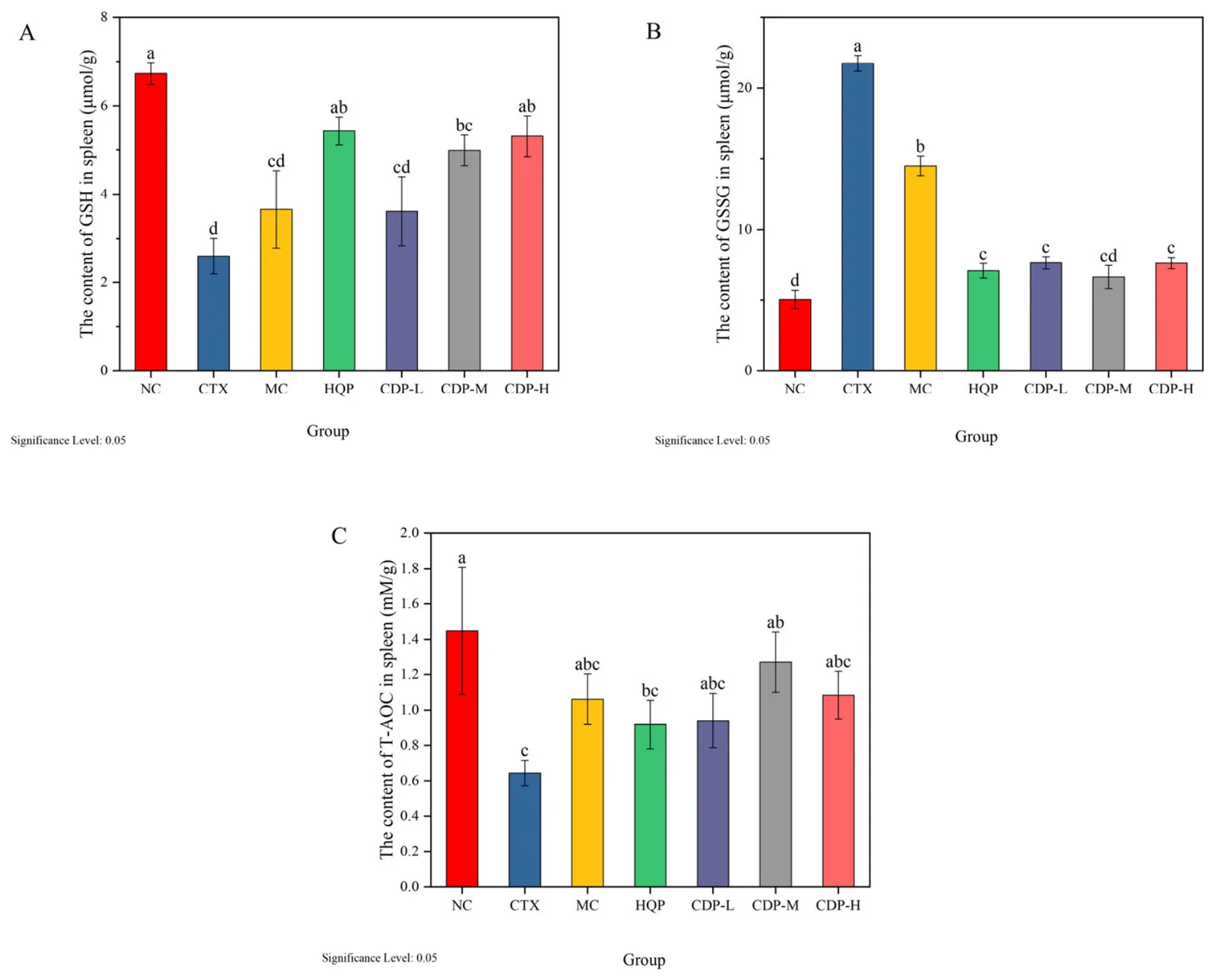
Effects of CDP on GSH (**A**), GSSG (**B**) and T-AOC (**C**) contents in spleen of immunosuppressed mice. Groups sharing the same letter(s) show no significant difference, while groups marked with different letters indicate a statistically significant difference (*p* < 0.05).

As shown in Figure 3A,B, CTX treatment led to an increase in the content of peroxidation products in the spleen, and the body adapted by increasing the content of GSH-Px in order to achieve homeostasis; the increase in GSH-Px prompted a concomitant increase in the content of GSSG, and the contents of GSH-Px and GSSG in the spleen of mice in the model group were significantly higher than those in the other groups (*p* < 0.05). GSH is the predominant antioxidant in the spleen. It maintains intracellular redox homeostasis by directly scavenging ROS (e.g., H_2_O_2_ and -OH) and reduced oxidative glutathione (GSSG). GSSG is a product of the oxidation of GSH by ROS, and its elevated level reflects the intensification of oxidative stress in the spleen.

#### 3.4.4. Effects on Content of T-AOC in Spleen of Mice

As can be seen in Figure 3C, compared with T-AOC in the spleen of mice in the model group, T-AOC was elevated in all other groups, and the difference in T-AOC content in the spleen of mice was significant only in the medium-dose-CDP-gavaged mice (*p* < 0.05). CTX metabolites directly deplete antioxidant enzymes (e.g., GSH-Px) and reduce T-AOC; meanwhile, they inhibit the Nrf2 pathway and block antioxidant gene expression, which results in increased splenic ROS levels and imbalanced CD4^+^/CD8^+^ T-cell ratios (decreased Th1 and increased Th2).

### 3.5. Effects of CDP Gavage on Pathology of Spleen Tissue in Immunologically Injured Mice

**Figure 4:**
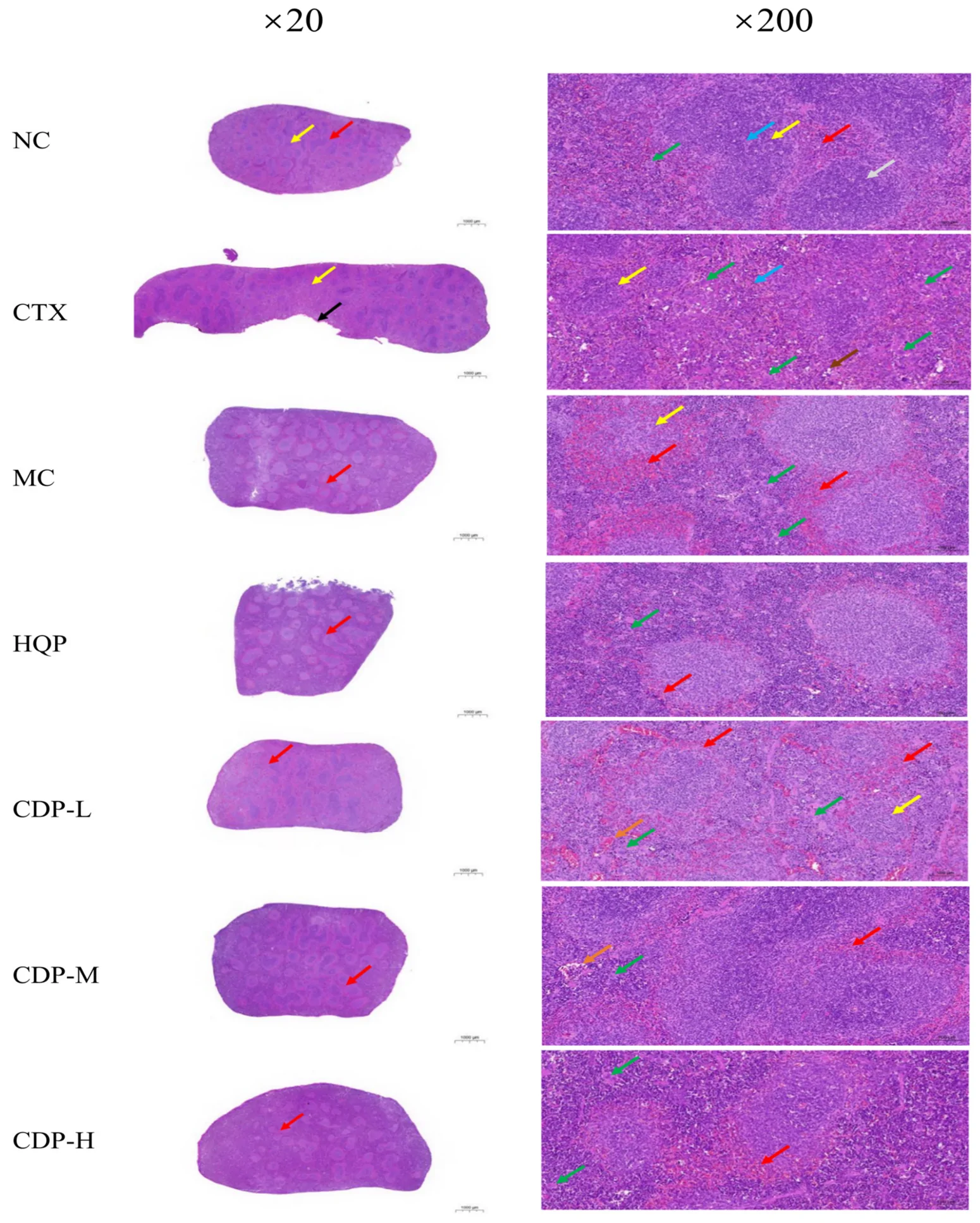
Pathological analysis of spleen tissue.

As can be seen in Figure 4, in NC mice, the peritoneum of the spleen consisted of dense connective tissue rich in elastic and smooth muscle fibers of uniform thickness, and the peritoneal connective tissue extended into the spleen to form trabeculae, with no obvious abnormality. The splenetic parenchyma consisted of red medulla oblongata (red arrowheads), white medulla oblongata (yellow arrowheads), and the marginal zone. The white medulla oblongata was composed of lymph sheaths around the central artery (blue arrowhead) and lymphoid nodes (gray arrowhead); the red medulla oblongata was distributed in the vast area under the peritoneum, around the trabeculae and outside the marginal zone of the white medulla and consisted of the splenic cord and splenic haemitin, with an occasional scattered distribution of megakaryocytes (green arrowhead), all of which did not show any obvious abnormality. In the mice in the CTX group, small areas of splenic tissue had uneven peritoneum (black arrowhead), with areas presenting uneven thickness and thinness. The splenic parenchyma was composed of red medulla, white medulla and the marginal zone. The white medulla consisted of lymphatic sheaths around the central artery and lymphoid nodule microstructures, and small areas of white medulla were seen; in particular, in a small area, white medulla cells were reduced in volume and size (yellow arrowheads) and had an irregular shape. The red medulla was distributed in the sub-periosteal area, around the trabeculae, and in the outer part of the marginal zone of the white medulla; it consisted of the splenic cord and splenic sinusoids, with a uniform distribution and an unclear boundary with the white medulla; it presented a large number of megakaryocytes (green arrowheads), with occasional infiltration of granulocytes (blue arrowheads), and a small number of vacuoles in a scattered distribution (brown arrowhead). In MC mice, the periosteum was composed of dense connective tissue rich in elastic and smooth muscle fibers of uniform thickness, and the periosteum connective tissue extended into the spleen to form trabeculae, with no obvious abnormality. The parenchyma of the spleen consisted of red medulla, white medulla, and the marginal zone. The white medulla consisted of lymphatic sheaths around the central arteries and lymphatic microstructures. The red medulla oblongata was distributed under the peritoneum, around the trabeculae and in the outer part of the marginal zone of the white medulla oblongata and consisted of the splenic cord and splenic sinusoids, with a lot of bruises around the white medulla oblongata (red arrows) and a large number of megakaryocytes (green arrows); further, a small amount of white medulla oblongata had reduced volume (yellow arrow). Similarly, in HQP mice, the white medulla included the central artery, peripheral lymphatic sheaths and lymphoid microstructures; the red medulla was distributed in the sub-periosteal area, around the trabeculae and in the outer part of the marginal zone of the white medulla and consisted of the splenic cord and splenic sinusoids, with a large number of periosteum bruises (red arrows) and a large number of megakaryocytes (green arrowhead). In mice in the CDP-L group, the periosteum consisted of thick, thin, and uniformly dense connective tissue rich in elastic fibers and smooth muscle fibers; the connective tissue extended into the spleen to form trabeculae, with no obvious abnormality. The parenchyma of the spleen consisted of red medulla, white medulla, and the marginal zone. The white medulla consisted of lymphatic sheaths around the central artery and lymphatic microstructures; the red medulla was distributed in the sub-periosteal area, around the trabeculae, and in a vast area outside the marginal zone of the white medulla and consisted of the splenic cord and splenic hematological sinuses. A small amount of siltation around the white medulla (red arrowheads), a small amount of white medulla with a reduced volume (yellow arrowhead), and more siltation of hematological sinuses could be observed. A small amount of white medulla was reduced in size (yellow arrow), a large number of blood sinusoids were bruised and dilated (orange arrow), and a large number of megakaryocytes were seen (green arrows). In mice in the CDP-M group, the peritoneum of the spleen was composed of dense connective tissue rich in elastin fibers and smooth muscle fibers of uniform thickness, and the peritoneal connective tissue extended into the spleen to form trabeculae, with no obvious abnormality. The red medulla oblongata was distributed under the peritoneum, around the trabeculae and the outer part of the marginal zone of the white medulla oblongata and consisted of the splenic cord and splenic blood sinuses. A large amount of silt around the white medulla oblongata could be seen (red arrows), with rare dilatation of the blood sinuses (orange arrow) and a small number of megakaryocytes (green arrow). In mice in the CDP-H group, the peritoneum of the spleen consisted of thick and thin dense connective tissue rich in elastin fibers and smooth muscle fibers which extended into the spleen to form trabeculae, with no obvious abnormality.

After histopathological examination, spleen tissues were observed under HE staining. No obvious pathological changes were observed in the spleen of mice in the NC group. Compared with the spleen tissues of mice in the NC group, the spleen tissues of mice in the CTX group showed a decrease in the amount of white medulla oblongata and a decrease in its volume, a significant increase in the number of megakaryocytes in red medulla oblongata, and mild infiltration of inflammatory cells. Compared with mice in the NC group, mice in the CDP-L group showed an increase in the extent of splenic tissue bruising, a mild decrease in the volume of white marrow, and an increase in the number of erythrocytic megakaryocytes. Compared with the spleen tissues of mice in the CTX group, the spleen tissues of mice in the CDP-L group had a higher number of white myeloid cells, an increased range of bruising, a mild decrease in the number of red myeloid megakaryocytes, and a decreased range of inflammatory cell infiltration. The spleen tissues of CDP-M and CDP-H mice had a higher number of white myeloid cells, an increased range of bruising, a significant decrease in the number of red myeloid megakaryocytes, and a decreased range of inflammatory cell infiltration; compared with the spleen tissues of CDP-L mice, the spleen tissues of CDP-M and CDP-H mice had a larger volume of white myeloid cells and a significantly reduced number of red myeloid megakaryocytes; the difference between the spleen tissues of CDP-M and CDP-H mice was not obvious. Compared with the spleen tissues of mice in the NC group, the spleen tissues of mice in the MC group showed a decrease in the volume of white myeloid cells, an increase in the extent of bruising, and an increase in the number of erythrocyte megakaryocytes; the spleen tissues of mice in the HQP group showed an increase in the extent of bruising and an increase in the number of erythrocyte megakaryocytes. Compared with the spleen tissues of mice in the CTX group, the spleen tissues of mice in the MC and HQP groups showed a larger volume and higher number of white medulla cells, and the number of erythrocyte megakaryocytes was mildly reduced. Compared with the spleen tissues of mice in the HQP group, the spleen tissues of mice in the CDP-L group were not significantly different; the spleen tissues of mice in the CDP-M and CDP-H groups had a larger volume and higher number of white myeloid cells, and the number of megakaryocytes in the red myeloid cells was significantly reduced. Compared with the spleen tissues of mice in the HQP group, the volume of white marrow cells in the spleen tissues of CDP-L mice was smaller, and other differences were not obvious; the number of red marrow megakaryocytes in the spleen tissues of CDP-M and CDP-H mice was reduced. CDP partially restored the spleen structure of immune-injured mice, the number of white marrow cells in the spleen tissues of mice rebounded, the number of red marrow megakaryocytes was reduced, the scope of inflammatory cell infiltration caused by CTX was reduced, and macrophage activity was increased.

### 3.6. Transcriptomic Data Analysis

The clean read data for all samples were identical to the raw read data, indicating that no data were lost during the filtering process and that the raw data were of high quality. Through transcriptomic sequencing, a total of 50,369,962 to 68,697,274 high-quality clean read sequences were obtained from samples across the different treatment groups. The base error rate was 0.02%, with Q20 and Q30 values both exceeding 98%. The GC content (49.55–50.43%) met the requirements. Overall, this indicates that the quality control of the sequencing data was satisfactory and that the data were suitable for subsequent analysis. A summary of the data is presented in Table 1.

**Figure 5:**
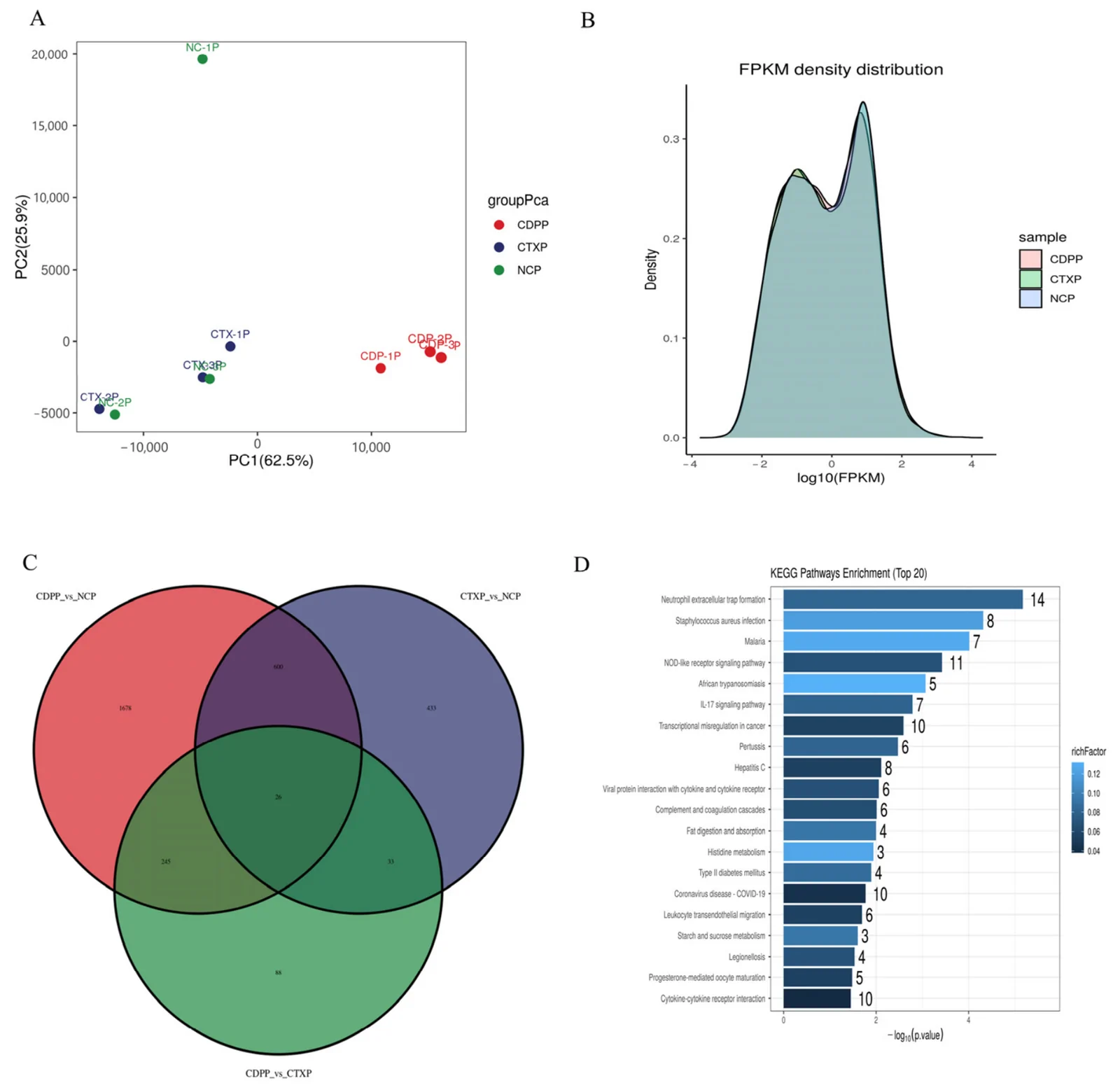
(**A**) PCA plot; (**B**) FPKM plots for each sample group; (**C**) Venn diagram of transcriptionally regulated genes; (**D**) KEGG enriched terms for the CDPP vs. CTXP group.

We assessed inter-group differences and the reproducibility of samples within groups. As shown in Figure 5A, the core direction of variation between samples is primarily concentrated on the PC1 (62.5%) dimension, and PC2 (25.9%), although lower than PC1, remains significantly higher than subsequent principal components. The CDPP and CTXP groups show clear separation along the PC1 axis, indicating core biological differences between the two groups. The CDPP group exhibits high clustering and good reproducibility, whilst the principal component distribution of the CTXP group is relatively concentrated. Although there are a few outlier samples, as their deviation direction (PC2) is unrelated to the core inter-group differences (PC1), the overall trends and conclusions of the data remain reliable. The NCP group exhibited better overall reproducibility, whilst the NC-1P group also demonstrated good overall reproducibility.

In the FPKM density plot (Figure 5B), the measured FPKM values for the genes are primarily distributed within the range of 10^−3^ to 10^3^, with gene expression across all sequenced samples exhibiting a clear tendency towards a concentrated distribution. The peak of each group’s curve is located at log10(FPKM) ≈ 0.2; the peaks are narrow and symmetrical, with a short tail on the left, indicating that most genes are at a moderately low expression level and that there are few low-expression genes.

The deviation in the NC-1P group may be attributed to technical errors or biological heterogeneity; however, as reproducibility within the other sample groups is high, this does not affect the reliability of the overall data trends or inter-group differences, and reproducibility between parallel samples is relatively good. As shown in the Venn diagram (Figure 5C), a total of 3103 genes were found to be differentially expressed among the different treatment groups. In the comparisons CDPP vs. NCP, CTXP vs. NCP and CDPP vs. CTXP groups, there were 26 genes common to all three, with 1678, 433 and 88 group-specific genes, respectively. The CDPP vs. CTXP comparison and the CDPP vs. NCP comparison exhibited 121 and 2278 differentially expressed genes, respectively, with 271 overlapping genes. KEGG overlay (Figure 5D) highlighted neutrophil extracellular trap (NET) formation (14 DEGs), NOD-like receptor (11 DEGs), and IL-17 signaling as CDPP-distinctive hubs, flanked by complement–coagulation crosstalk, leukocyte trans-endothelial migration, and generic Staphylococcus/viral-associated gene sets (adjusted *p* < 0.05). Rather than inferring broad-spectrum anti-infection efficacy, this multi-pathway convergence defines a transcriptional preparedness profile biased toward neutrophil-mediated containment and barrier immunity. CDPP thus layers innate-priming atop baseline CTXP-induced immune resetting without overstatement of translational scope.

**Figure 6:**
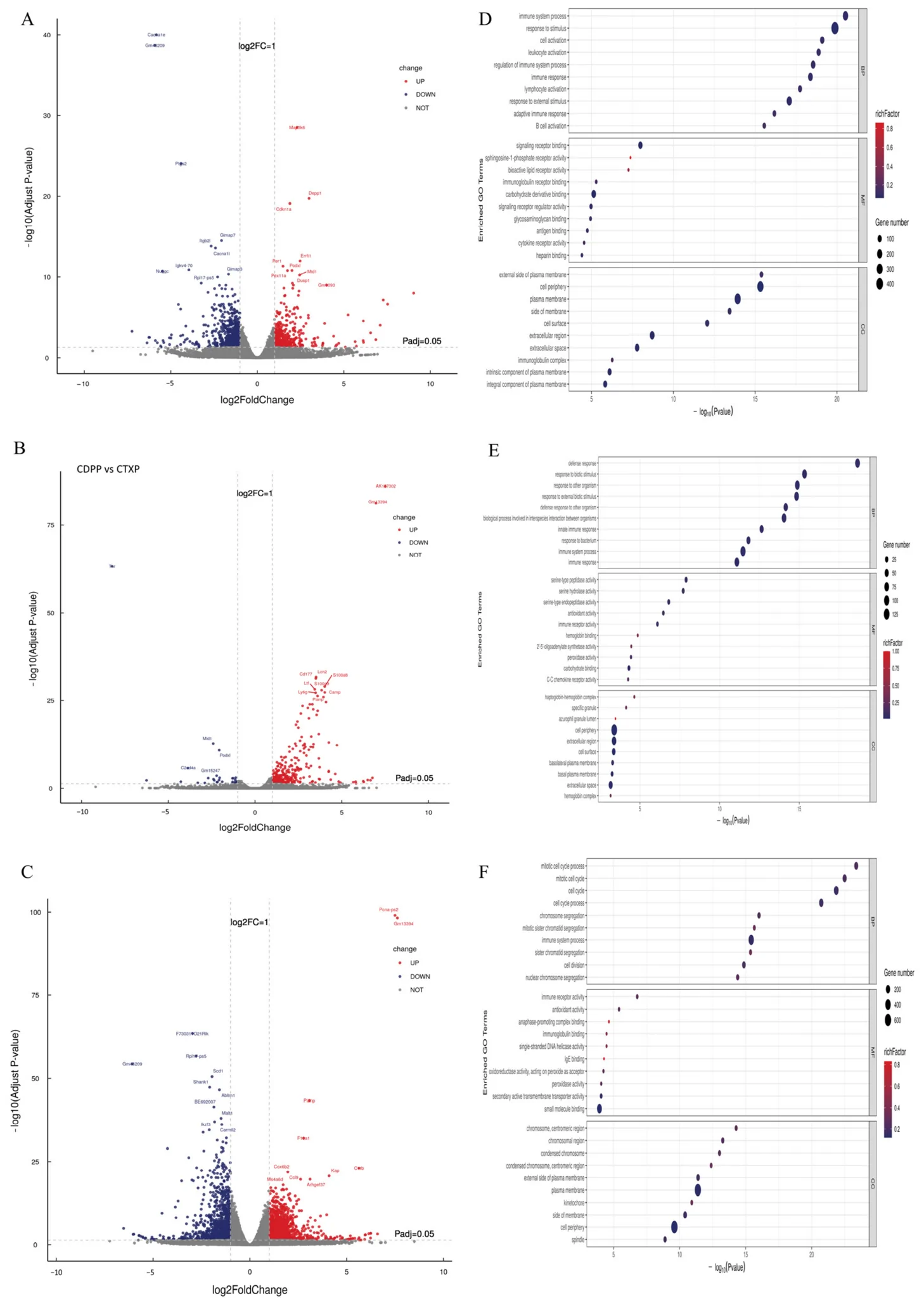
Volcano plots of differentially expressed genes in the NCP, CTXP and CDPP groups ((**A**) CTXP vs. NCP; (**B**) CDPP vs. CTXP; (**C**) CDPP vs. NCP) and GO enrichment bubble plots. (**D**) CTXP vs. NCP; (**E**) CDPP vs. CTXP; (**F**) CDPP vs. NCP.

Figure 6A–C show that 392 differentially expressed genes were identified between the CDPP and CTXP groups (360 up-regulated and 32 down-regulated), whilst 2651 were identified between the CTXP and NCP groups (1549 up-regulated and 1102 down-regulated), and 2549 were identified in the comparison between the CDPP and NCP groups (1402 up-regulated and 1147 down-regulated). These results indicate that compared with the NC group, gene expression in immunodeficient mice undergoes significant changes, and their transcriptional expression levels may be regulated by CDP.

Directional GO enrichment mapped CTXP-dominant DEGs to three functional strata without inferring causality. Biological process (BP) terms clustered around adaptive-effector modules—leukocyte/lymphocyte activation, B-cell humoral pathways, and antigen-driven immune response—while molecular function (MF) peaks centered on antigen/immunoglobulin binding, cytokine/lipid receptor activity, and glycosaminoglycan recognition. This signature parallels the quantitative restoration of splenic white-pulp follicles in CTXP-treated mice, framing CTXP as permissive to conventional humoral rebound without invoking mechanistic overstatement. As shown in Figure 6D,E, the GO enrichment analysis for the CTXP vs. NCP group revealed that BP processes, including immune system processes, cell, leukocyte and lymphocyte activation, adaptive immune response, and B-cell activation pathways, were highly significant. This indicates that following CTXP intervention, immune activation and adaptive immune responses in the body were extensively activated, particularly B-cell-related humoral immune responses and the activation processes of leukocytes or lymphocytes. MF encompasses functions such as antigen binding, immunoglobulin receptor binding, cytokine receptor activity, sphingosine-1-phosphate receptor and bioactive lipid receptor activity, and glycosaminoglycan/carbohydrate derivative binding. Among these, the enrichment fold for sphingosine-1-phosphate receptor activity was the highest, reflecting the important role of lipid receptor signaling, immune receptor recognition and cytokine signal transduction in this process [22].

The GO mapping of DEGs (FDR < 0.05) delineated a transition in regulatory emphasis between treatments. Relative to NCP, CTXP enriched adaptive-linked compartments (e.g., plasma membrane, immunoglobulin complexes, and extracellular space), consistent with humoral-effector gene deployment. Compared with CTXP, CDPP shifted enrichment to innate-biased signatures: biologic processes (BPs) of pathogen/sterile inflammatory response, serine-protease cascades, and oxidant handling; molecular functions (MFs) centered on immune receptor/chemokine binding and peroxidase activity; cellular components (CCs) on extracellular granules and membrane-proximal zones. Cell-cycle/BP terms (e.g., mitotic segregation and spindle organization) were uniquely prominent in CDPP vs. NCP, aligned with restored splenic white-pulp cellularity, framing CDPP as coordinately modulating proliferation, redox tone, and innate-effector output.

### 3.7. Proteomic Analysis

**Figure 7:**
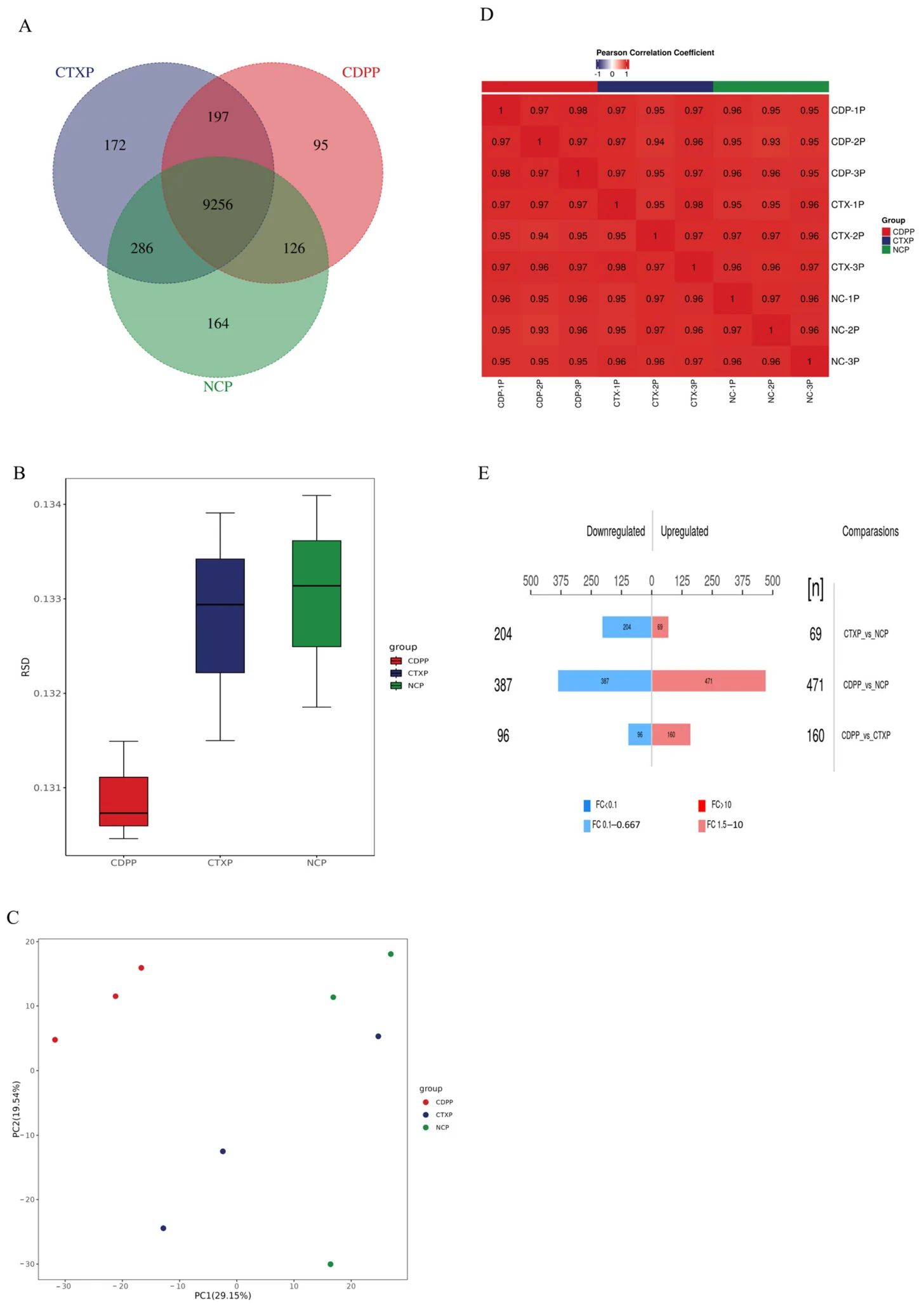
(**A**) Venn diagram summarizing protein identification results; (**B**) RSD box-and-whisker plot; (**C**) 2D and 3D principal component analysis plots; (**D**) Pearson correlation coefficient; (**E**) bar chart of differentially expressed proteins.

As shown in Figure 7A, 9832, 9911 and 9674 differentially expressed proteins were identified in the NCP, CTXP and CDPP groups, respectively. A total of 9256 differentially expressed proteins were common to all groups, accounting for the vast majority of the differentially expressed proteins across the groups. The number of differentially expressed proteins unique to the NCP and CDPP groups was lower than that in the CTXP group. This indicates that CTXP treatment induces more specific changes in protein expression, whereas NCP and CDPP treatments result in fewer unique protein alterations; their effects may be more evident in pathways that overlap with those of CTXP. As shown in Figure 7B, the RSD values for all groups are less than 20%, indicating good reproducibility of the quantitative results across samples, which is sufficient for subsequent data analysis [23].

The PCA results are shown in Figure 7C; the closer the samples are clustered in the figure, the more similar their protein expression profiles are. Comparing the samples within each group revealed that the degree of clustering in the CDPP group differed significantly from that in the CTXP and NC groups, indicating that the CDPP samples exhibited the highest similarity. The CTXP and NC groups, in turn, demonstrated variability both between and within sample groups. Pearson correlation analysis (Figure 7D) indicates that as the correlation coefficient approaches 1, the degree of positive correlation between samples is considered to be stronger. Within each experimental group, the correlation coefficients between replicate samples were observed to exceed 0.9, providing ample evidence of good intra-group sample reproducibility. This demonstrates that these samples exhibit a high degree of similarity in gene expression patterns, thereby ensuring the stability and reliability of the experimental results. Through comparative analysis of differentially expressed proteins in the proteomic sequencing data from the three groups (Figure 7E), a total of 1387 differentially expressed proteins were identified, of which 700 were up-regulated and 687 were down-regulated. In the CDPP vs. CTXP comparison, 160 proteins were up-regulated, and 96 were down-regulated. In the CTXP vs. NCP comparison, 69 proteins were up-regulated, and 204 were down-regulated. This suggests that CDP may exert its effects through additional mechanisms to reverse the molecular alterations induced by cyclophosphamide.

**Figure 8:**
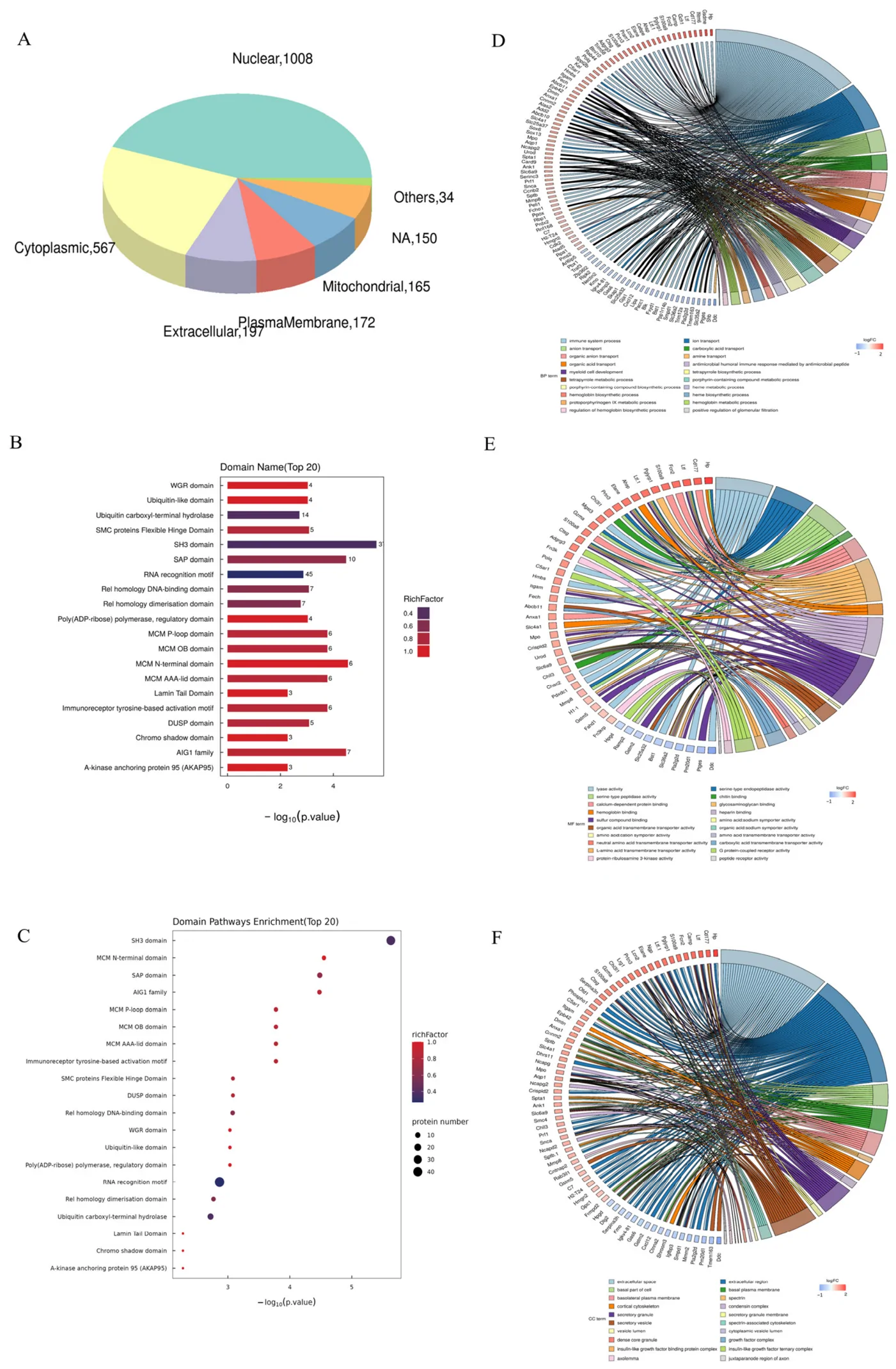
(**A**) Pie chart showing the subcellular localization of differentially expressed proteins. (**B**) Bar chart showing the predicted domains of differentially expressed proteins. (**C**) Diagram showing domain enrichment analysis of differentially expressed proteins. (**D**) Biological process (BP) chord diagram highlighting enriched terms linked to myeloid activation and innate immune defense. (**E**) Molecular function (MF) chord diagram illustrating calcium/heme binding and serine-protease cascades. (**F**) Cellular component (CC) chord diagram showing secretory granule and cytoskeletal topology.

Subcellular prediction localized the bulk of quantified proteins to the nucleus and cytoplasm, with minorities assigned to the plasma membrane, mitochondria, and extracellular space (Figure 8A). Domain-overrepresentation profiling (Figure 8B,C) identified RNA recognition motifs and MCM replicative helicase modules as dominant nucleocytoplasmic features, accompanied by deubiquitinate/ubiquitin-like domains and Rel-homology/ITAM-like signatures. Rather than recapitulating generic domain biology, this repertoire signals concurrent demand for post-transcriptional processing, replication licensing, and proteostatic turnover under the experimental regime.

Group-wise differential annotation ranked immune system process and myeloid-cell development as top biological process (BP) terms (Figure 8D), flanked by heme/porphyrin biosynthesis and organic-anion transport. Molecular function mapping emphasized serine-type peptidase activity, consistent with extracellular proteolysis in innate immunity. Ion/organic-acid transporter hits aligned with porphyrin-metabolism terms, sketching a stress-adaptive proteome balancing redox load and secretory immunity. No causal attribution to specific signaling cascades is inferred here; comparative domain shifts between CDPP and CTXP simply define a distinct molecular signature awaiting functional validation [24].

GO annotation delineated functional topology without mechanistic extrapolation. Molecular function (MF) (Figure 8E) hits clustered into four non-redundant themes: (i) catalytic/serine-type peptidase activity; (ii) calcium/hemoprotein binding—consistent with established splenic stress–proteome annotations [25,26,27]; (iii) sulfur–compound interaction (glutathione/methionine pools), denoting redox-handling capacity; (iv) organic-acid/L-amino acid transmembrane transport, reflecting substrate flux for immune-cell metabolism. Ribulose-bisphosphate kinase registered minimally, indicating narrow enzymatic representation.

Cellular component (CC) mapping (Figure 8F) allocated proteins to cytoskeletal/cortical actin, secretory/dense-core granules, extracellular niche, and basolateral membrane zones, with sparse axonal/myelin tagging [28,29,30,31]. Cytoskeletal enrichment parallels myeloid-cell migratory/effector potential, granule accumulation aligns with restored splenic white-pulp volume, and transport–binding modules frame homeostatic adaptation without inferring lineage-specific functions.

**Figure 9:**
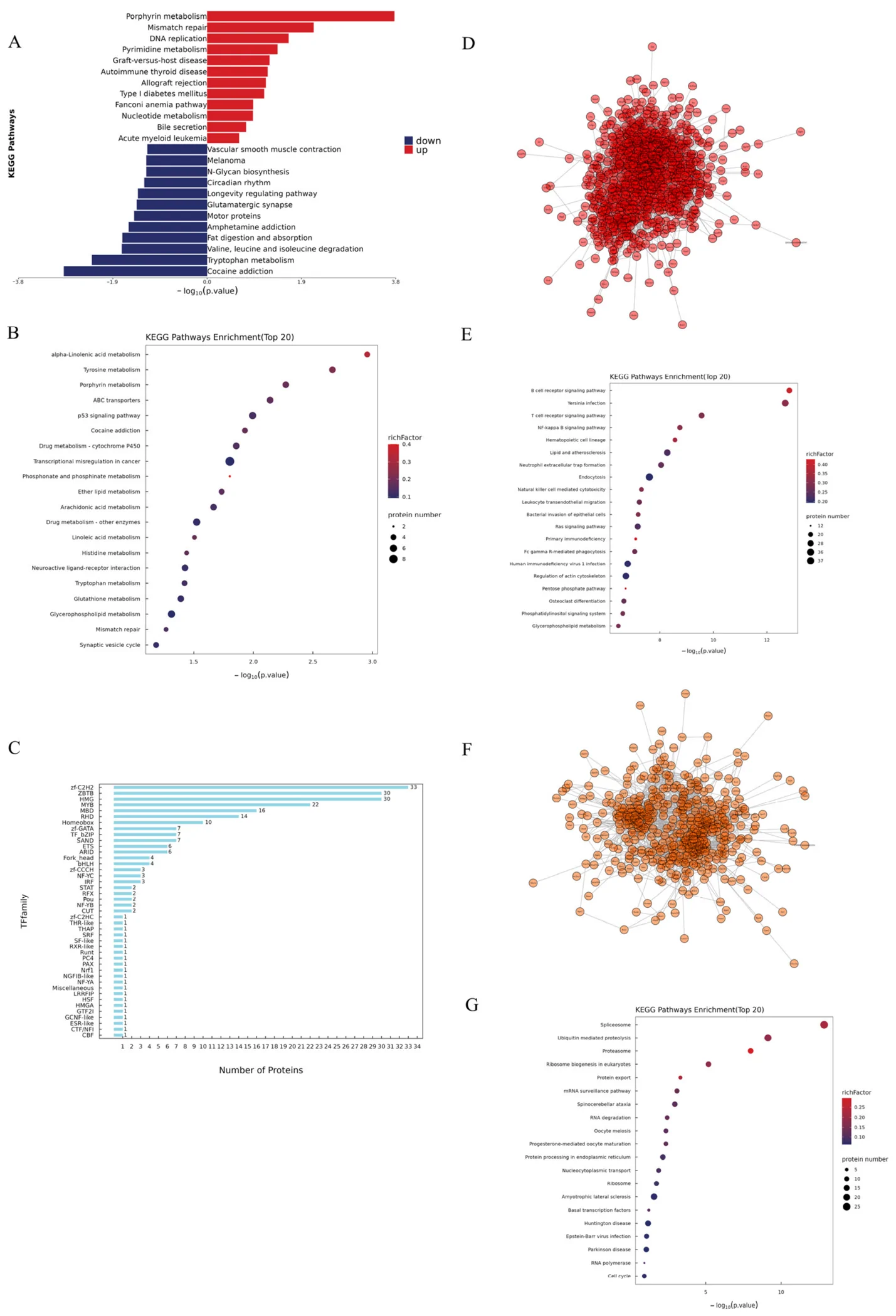
(**A**) Pathway enrichment butterfly plot of up- and down-regulated differentially expressed proteins between the CDPP and CTXP groups. (**B**) KEGG pathway enrichment bubble plot of differentially expressed proteins between the CDPP and CTXP groups. (**C**) Transcriptional factor analysis. (**D**) Cluster_1 PPI analysis. (**E**) Cluster_1 KEGG enrichment analysis. (**F**) Cluster_2 PPI analysis. (**G**) Cluster_2 KEGG enrichment analysis.

KEGG enrichment of DEPs (Figure 9A,B) showed directional up-regulation of immune-associated modules (e.g., allograft rejection, autoimmune thyroid disease, and type I diabetes) contrasted by down-regulation of amino acid catabolism and tryptophan metabolism. Ranked by –log_10_(p), top hits were α-linolenic acid, tyrosine/porphyrin metabolism, ABC transporters, and p53 signaling; immune pathways predominated numerically, and metabolic terms carried tighter per-pathway significance. These shifts paralleled quantitative splenic histopathology showing restored white-pulp area in CDPP.

TF domain mapping (Figure 9C) allocated 237 DEPs to 43 families, led by zinc-finger (e.g., zf-C2H2 and ZBTB) and HMG boxes, with minor representation of stress (STAT/HSF) and lymphoid (ETS/IRF) regulators. Multi-domain scaffolds denoted architectural complexity without inferring lineage commitment.

PPI clustering resolved two non-redundant modules (Figure 9D–G): Cluster 1 enriched B/T-cell, NOD-like receptor, FcγR, and NF-κB/MAPK/PI3K nodes; Cluster 2 centered on spliceosome, ribosome biogenesis, ubiquitin-proteasome, and ER processing. The bipartite topology describes resource partitioning between immune signaling and proteostatic support—strictly a correlative organizational pattern pending functional validation.

### 3.8. Integrated Transcriptomic and Proteomic Analyses

**Figure 10:**
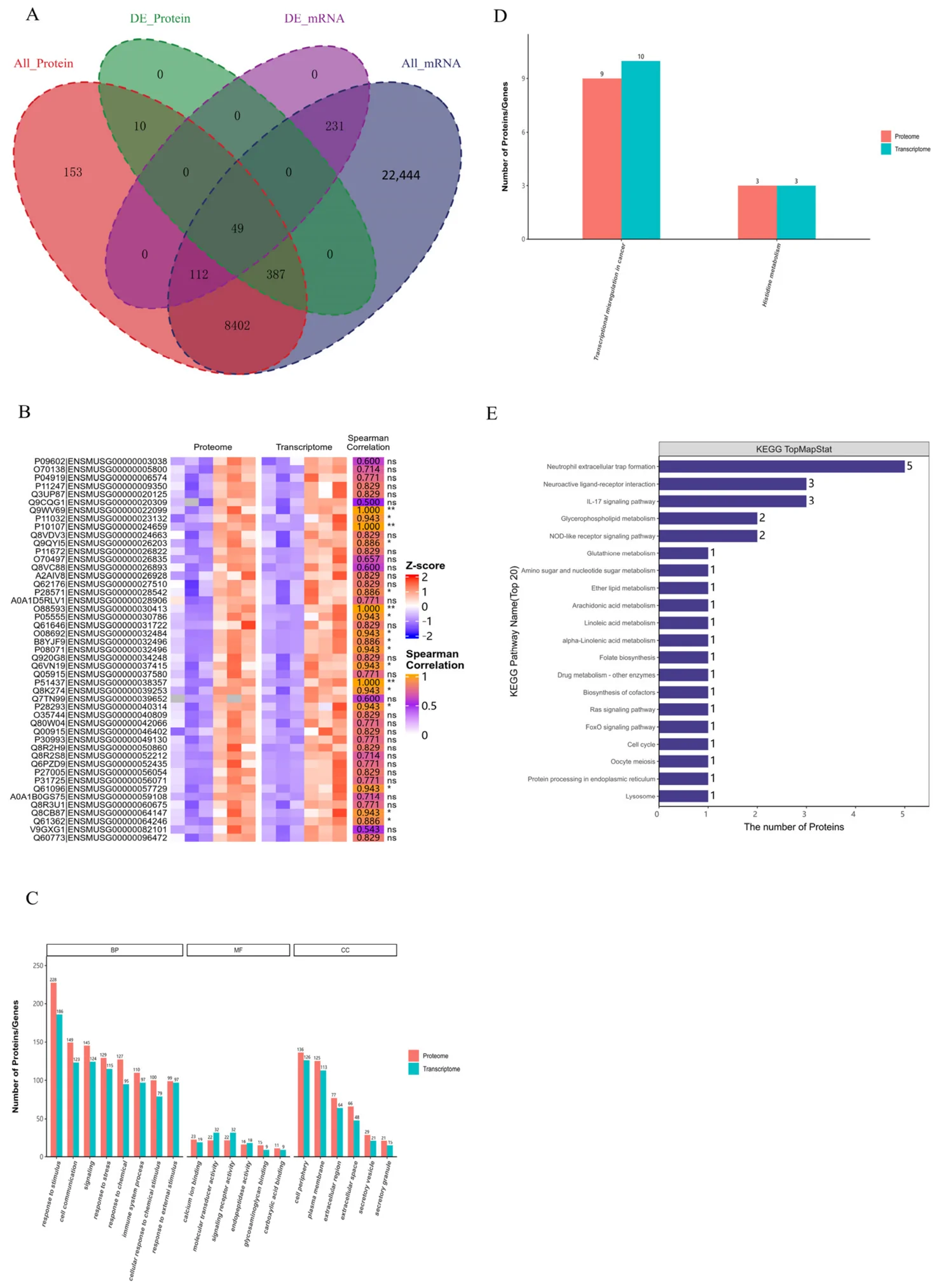
(**A**) Venn diagram showing the number of associations between the CDPP and CTXP groups. (**B**) Association analysis of differentially expressed genes and proteins. (**C**) Enriched GO terms. (**D**) Enriched KEGG pathways. (**E**) KEGG pathways by number of differentially expressed proteins. Asterisks indicate statistically significant correlations (* *p* < 0.05, ** *p* < 0.01). ns: not significant (*p* ≥ 0.05).

Quantitative profiling identified 9650 quantifiable proteins and 31,625 genes across groups, with 9498 proteins and 8950 transcripts forming overlapping detection sets (Figure 10A). By applying thresholds of |log_2_FC| > 1 and adj. *p* < 0.05, we detected 453 differentially expressed proteins (DEPs) and 392 differentially expressed genes (DEGs), of which 50 DEPs and 49 DEGs constituted a directly associated co-regulated set (Figure 10B). Spearman correlation analysis revealed predominantly positive mRNA–protein concordance in the CDPP vs. NCP comparison, with several pairs reaching nominal significance (*p* < 0.05; e.g., antimicrobial peptide P51437 and immune modulator O88593; *p* < 0.01 for glycine transporter P28571 and RNA-processing factor Q9WV69), suggesting predominant transcriptional-level regulation rather than extensive post-translational buffering for these candidates.

GO enrichment of the CDPP vs. CTXP signature prioritized biological processes (BPs) of stress response (228 DEPs/186 DEGs) and cell–cell communication (149 DEPs/123 DEGs), followed by immune system process and signal transduction (Figure 10C). Molecular function (MF) terms clustered around Ca^2+^ binding and signal receptor activity, while cellular component (CC) mapping localized hits to the plasma membrane, extracellular region, and secretory vesicles—collectively defining a secretory, stress-responsive phenotype without inferring functional causality at this stage.

Pathway-level convergence was the clearest for Transcriptional dysregulation in cancer (9 DEPs/10 DEGs), reflecting broad transcriptional network remodeling. By contrast, histidine metabolism showed minimal shared hits (three molecules), indicating secondary metabolic flux rather than primary targeting. KEGG mapping highlighted neutrophil extracellular trap (NET) formation (map04613) as the most enriched proteomic pathway (5 DEPs), flanked by IL-17 signaling and NOD-like receptor cascades (3 DEPs each), with glycerophospholipid metabolism and glutathione pathways as minor contributors (≤2 DEPs) (Figure 10D,E).

Network projection onto the NET-formation schema placed DEPs at upstream pattern-recognition receptors (e.g., TLR2/4 and complement receptor), intracellular PI3K-Akt/MAPK nodes, and effector modules (e.g., MPO, NE, and PAD4-linked chromatin modification). No cytological NET validation was attempted here; however, the multi-omics overlay consistently flags innate-effector priming as a distinctive molecular fingerprint of CDPP relative to CTXP rescue alone.

## 4. Discussion

Common cytokines are mainly autocrine and paracrine, binding to specific receptors on the surface of target cells, triggering cell proliferation and differentiation, inflammatory response and immune response, and enhancing the body’s ability to eliminate and resist pathogens [32]. Th1 cells secrete cytokines, such as INF-γ and TNF-α, which mediate cellular immunity; Th2 cells secrete IL-1 and IL-6, which mediate humoral immunity, and the two synergistically enhance the immune response of the organism [33]. The humoral and cellular immune status of the organism can be reflected by measuring the serum levels of IFN-γ, TNF-α and IL-6. The immunomodulation of CDP was shown to be dose-dependent, with the medium-dose group showing the best results in terms of spleen index recovery and improvement in antioxidant indices, whereas the inhibitory effect on pro-inflammatory factors was more pronounced in the high-dose group. This non-linear effect may be related to the saturation binding properties of polysaccharide receptors.

The results show that CDP significantly elevated the splenic index in immunosuppressed mice, restored the splenic histological structure, and regulated the expression levels of key cytokines (TNF-α, IFN-γ, IL-1β, IL-2, and IL-6). These findings are consistent with existing studies, for example, Han et al. [34] found that hawthorn leaf polysaccharides activated dendritic cell function through the TLR4/NF-κB pathway, whereas in the present study, CDP showed a more comprehensive immunomodulatory capacity by regulating the Th1/Th2 balance and the ratio of pro-inflammatory/anti-inflammatory factors. Notably, the up-regulation of IL-2 and inhibition of IL-6 by CDP suggest that it may improve the immunosuppressive state by modulating T-cell activation and B-cell differentiation pathways.

Oxidative stress is an important mechanism of CTX-induced immune injury. In this study, we found that CDP significantly reduced MDA and ROS levels in the spleen while elevating the activities of antioxidant enzymes such as GSH-Px, GR, and SOD. This result bears similarity to the study of polysaccharides from young barley leaves by Jiang et al. [7]. However, CDP was more prominent in restoring GSH/GSSG balance. Notably, the bidirectional regulation of CAT activity by CDP suggests that it may dynamically regulate the oxidative stress response through the Nrf2-Keap1 pathway. The observed SOD “overshoot” alongside partial CAT recovery is mechanistically plausible: exogenous polysaccharides act as sacrificial radical scavengers, curtailing H_2_O_2_ flux and relaxing selective pressure on catalase whilst sustaining SOD to intercept residual superoxide leakage. Such non-parallel antioxidant enzyme trajectories are reported for botanical glycans and reflect adaptive enzymatic stoichiometry rather than failed rescue. Thus, CDP remodels redox tone via a targeted enzymatic bias, not blanket antioxidant system elevation. However, the dose-dependent increase in T-AOC further confirmed that CDP protected splenic immune cells from oxidative damage by enhancing the overall antioxidant capacity, thereby maintaining T-cell receptor (TCR) signaling and B-cell antibody production functions.

Transcriptomic analysis revealed that the sequencing data were of high quality and exhibited good reproducibility, with significant core biological differences observed between the CDPP, CTXP and NCP groups. Analysis of differentially expressed genes indicated that CTXP intervention primarily induced systemic immune activation centered on adaptive immune responses, whereas CDPP intervention, building upon the CTXP model, significantly modulated immune inflammation and stress responses by activating innate immune defenses, anti-infection pathways and antioxidant protective mechanisms. Furthermore, compared with NCP, it demonstrated synergistic regulation of the cell cycle, immune activation and oxidative stress balance. In-depth KEGG pathway analysis of the CDPP vs. CTXP groups revealed that CDPP can, via core pathways including neutrophil extracellular trap formation, NOD-like receptor and IL-17 signaling, and the complement–coagulation cascade, construct a regulatory network centered on innate immune activation, pathogen clearance, and inflammatory microenvironment homeostasis. This demonstrates a more targeted advantage in anti-infective and immune regulation compared with CTXP, providing a crucial molecular basis for subsequent mechanistic validation and translational research. CDPP maintains the immune-inflammatory balance by ‘precisely regulating’ innate immune pathways. Similarly, the NOD-like receptor protein (NLRP) family is a key component of the NOD-like receptor (NLR) superfamily and plays a crucial role in regulating apoptosis, triggering inflammatory responses and mediating innate immunity [35]. Inflammasomes are innate immune complexes capable of recognizing a wide range of pathogens, cellular stress and damage signals; they also trigger the production of Caspase-1-dependent inflammatory cytokines, including IL-1β and IL-18 [36]. Dysregulation of the NOD-like receptor protein 3 inflammasome is one of the core mechanisms underlying the pathogenesis of various inflammatory diseases, such as arthritis, Alzheimer’s disease and inflammatory bowel disease [37]. IL-17 is not only a key cytokine in the host’s defense against mucosal infections but also a key therapeutic target for numerous autoimmune and chronic inflammatory diseases [38]. Th17 cells possess distinct transcription factors, including RORγ, RORα, IRF-4 and AhR, which are involved in cell lineage-specific differentiation and cytokine production [39]. Among the numerous studies currently underway, Li et al. have investigated the mechanism by which anti-complement heteropolysaccharide (HCPM) derived from Houttuynia cordata alleviates lung injury caused by the influenza A virus (H1N1). They found that this polysaccharide exerts a synergistic immunomodulatory effect by inhibiting the activation of the NLRP3 inflammasome and regulating the Treg/Th17 balance, demonstrating its significant potential as a multi-targeted therapeutic agent in severe infections [40]. Shi et al. investigated the potential of Houttuynia cordata polysaccharide (HCP) to treat viral lung infections via oral administration. They found that HCP acts on the intestinal mucosal immune system to modulate the balance between Th17 cells (which produce IL-17) and Treg cells, subsequently influencing infection and damage in the lungs via the CCR6/CCL20 axis [41]. This provides a molecular basis for the systemic anti-infective effects of polysaccharides, which are achieved by remotely regulating the balance of key immune-cell subsets in the body.

Proteome-wide GO/KEGG mapping reinforced this transcriptional signature without overstating causality. BP terms prioritized myeloid development, ion/organic-acid transport, and heme handling, while MF hits converged on serine-protease cascades, calcium binding, and redox-active sulfur–compound interactions—a biochemical ensemble compatible with CTX-induced stress adaptation rather than de novo pathway discovery [25]. CC allocation to secretory granules, cortical actin, and sparse axonal annotations was noted; however, we refrain from inferring neuroimmune cognitive roles, framing these merely as a motile, secretory proteome topology.

KEGG stratification echoed porphyrin/tyrosine flux, ABC transporter efflux, and p53-node stress integration—findings aligned with established genotoxic/oxidative signatures of alkylating agents. This cluster exhibits characteristics driven by immune inflammation, widespread activation of signaling pathways, and coordinated responses in metabolic and cellular processes [42]. TF domain parsing and PPI modularity resolved two non-redundant clusters: Cluster 1 enriched B/T-cell and NOD-like receptor/NF-κB nodes, and Cluster 2 centered on spliceosome/ribosome/ubiquitin proteasome. Wu et al. systematically summarized how metabolite-driven post-translational modifications link metabolic states to immune regulation; metabolite-driven (metabolite-derived post-translational modifications) PTMs play a key role in regulating immune networks, offering new avenues for the treatment of cancer, autoimmune diseases and inflammatory conditions [43]. Their co-enrichment describes a stoichiometric resource-partitioning model wherein immune activation is scaffolded by basal gene-expression machinery, not a novel regulatory hierarchy per se.

Multi-omics convergence affirmed predominant transcriptional-level control, with CDPP layering innate programs—NET-formation proxies, IL-17/NOD signaling—atop CTXP’s humoral reset. We emphasize that NET-associated markers reflect putative transcriptional priming pending cytological validation. Collectively, CDP engages a pragmatic triage of innate signaling, redox buffering, and translational fidelity in the murine CTX model; extrapolation to broad-spectrum anti-infective therapy awaits targeted functional validation. While the proteomic screen identifies compelling innate-effector candidates, targeted validation was beyond the present study’s scope and will be addressed in forthcoming work to confirm translational relevance.

## 5. Conclusions

In this study, we systematically revealed the mechanism of action of CDP in repairing spleen function in immunosuppressed mice by integrating physiological indexes and transcriptomics techniques. CDP significantly reversed CTX-induced splenic atrophy, repaired the structural integrity of white medulla oblongata, and re-established Th1/Th2 immune homeostasis by dose-dependently modulating the IL-2/IL-6 ratio. By activating the GSH-Px/SOD antioxidant system, CDP effectively scavenges splenic ROS and MDA, improves the microenvironment of immune cells, and its GSH regeneration ability is significantly better than that of comparable Phyto polysaccharides. Results from transcriptomic, proteomic and multi-omics analyses indicate that the sequencing data are of reliable quality and exhibit good reproducibility, with significant core biological differences observed between the CDPP, CTXP and NCP groups. CDPP demonstrates clear systemic immunomodulatory advantages over CTXP; this intervention activates innate immune defense, anti-infection pathways and antioxidant protective mechanisms, effectively regulating immune inflammation and stress responses whilst achieving synergistic regulation of the cell cycle, immune activation and oxidative stress balance compared with NCP. Multi-omics profiling (CDPP vs. CTXP/NCP) confirmed high technical fidelity and delineated a CDP-distinctive signature: preferential transcriptional-level modulation of innate-effector proxies atop conserved stress–metabolism programs, rather than de novo pathway invention. Correlation analysis of sentinel effectors supported predominant transcriptional governance with limited post-translational decoupling. Collectively, CDP emerges as a murine splenic resilience modulator that couples innate immune priming; mechanistic depth beyond transcriptional association and translational utility in infection models warrant dedicated future validation.

## Figures and Tables

**Table 1:** Summary statistics of mouse spleen transcription.

Category	CDP			CTX			NC		
Sample Name	CDP-1P	CDP-2P	CDP-3P	CTX-1P	CTX-2P	CTX-3P	NC-1P	NC-2P	NC-3P
Raw_reads	50,369,962	61,119,104	60,364,154	53,725,692	58,515,588	68,697,274	64,105,622	57,087,344	63,991,264
Clean_reads	50,369,962	61,119,104	60,364,154	53,725,692	58,515,588	68,697,274	64,105,622	57,087,344	63,991,264
Clean_bases	7.51 G	9.11 G	9 G	8.01 G	8.73 G	10.23 G	9.57 G	8.48 G	9.54 G
Error(%)	0.02	0.02	0.02	0.02	0.02	0.02	0.02	0.02	0.02
Q20(%)	99.71	99.72	99.74	99.72	99.69	99.74	99.7	99.72	99.72
Q30(%)	98.86	98.89	98.93	98.9	98.75	98.96	98.8	98.91	98.89
GC(%)	50.19	50.16	50.13	49.89	50.01	49.83	50.43	49.55	49.91

## Data Availability

All data analyzed or generated during this study are included in this published article.
